# Evaluating trends in damage to attractive targeted sugar baits (ATSBs) deployed during the second year of a two-year Phase III trial in Western Zambia

**DOI:** 10.1186/s12936-024-05089-5

**Published:** 2024-08-29

**Authors:** Irene Kyomuhangi, Joshua Yukich, Kochelani Saili, Erica Orange, Mundia H. Masuzyo, Mwansa Mwenya, Patricia Mambo, Busiku Hamainza, Joe Wagman, John Miller, Javan Chanda, Kafula Silumbe, Megan Littrell, Thomas P. Eisele, Ruth A. Ashton

**Affiliations:** 1 Centre for Applied Malaria Research and Evaluation, Tulane School of Public Health and Tropical Medicine, New Orleans, LA USA; 2 Present Address: Centre for Health Informatics Computing and Statistics, Lancaster University, Lancaster, UK; 3 PATH, Seattle, WA USA; 4 PATH, Kaoma, Zambia; 5 Present Address: Macha Research Trust, Choma, Zambia; 6 National Malaria Elimination Centre, Lusaka, Zambia; 7 PATH, Lusaka, Zambia; 8 PATH, Washington DC, WA USA

**Keywords:** Malaria, Attractive targeted sugar bait, Vector control

## Abstract

**Background:**

Attractive Targeted Sugar Baits (ATSBs) are a proposed new vector control tool for malaria that contain sugar and an ingestion toxicant, and are designed to attract and kill sugar-feeding mosquitoes. During a two-arm cluster randomized Phase III trial conducted in Zambia to test the efficacy of ATSB stations on malaria incidence, ATSB stations deployed on eligible household structures within intervention clusters were routinely monitored to ensure their good physical condition and high coverage. This study investigates trends in prevalence and rate of damage to ATSB stations during year 2 of the two-year trial.

**Methods:**

The analysis was conducted using monitoring data collected in year 2, which included types of damage observed, location, and date of removal and/or replacement of ATSB stations. The study evaluated temporal trends in the prevalence of overall damage and different damage types among 68,299 ATSB stations deployed. A profile of all ATSB stations installed on each structure was constructed, and spatial analyses conducted on overall damage and different damage types observed on 18,890 structures. Mixed effects regression analyses were conducted to investigate drivers of damage to ATSB stations on these structures.

**Results:**

Prevalence of overall damage and different damage types was temporally and spatially heterogeneous. Among damaged ATSB stations observed during monitoring, tears and mold had the highest prevalences on average, with tears maintaining above 50.0% prevalence through most of the monitoring period, while mold prevalence increased steadily during the first few months, peaking in February. Overall, 45.6% of structures had at least one damaged ATSB station, however this varied spatially across the trial site. Both structure characteristics and environmental factors significantly impacted the odds and rate of damage to ATSB stations on structures, including: ATSB stations’ level of protection from rainfall and sunshine; roof and wall material of the structure; night-time temperature; rainfall; enhanced vegetation index, and land cover.

**Conclusion:**

Damage to ATSB stations in this setting was common and was temporally and spatially heterogeneous. This has implications on operational feasibility, sustainability, and cost of future deployment. Further research is required to understand the mechanisms of damage, and to minimize prevalence and rate of damage to ATSB stations.

**Supplementary Information:**

The online version contains supplementary material available at 10.1186/s12936-024-05089-5.

## Background

Malaria remains a significant global health concern with close to 250 million cases and 600,000 deaths estimated in 2022 [1]. While there have been significant gains in reducing the global malaria burden, progress has stalled, necessitating new innovative tools to meet the emerging threat of resurgence of this disease [1–3].

**Fig. 1:**
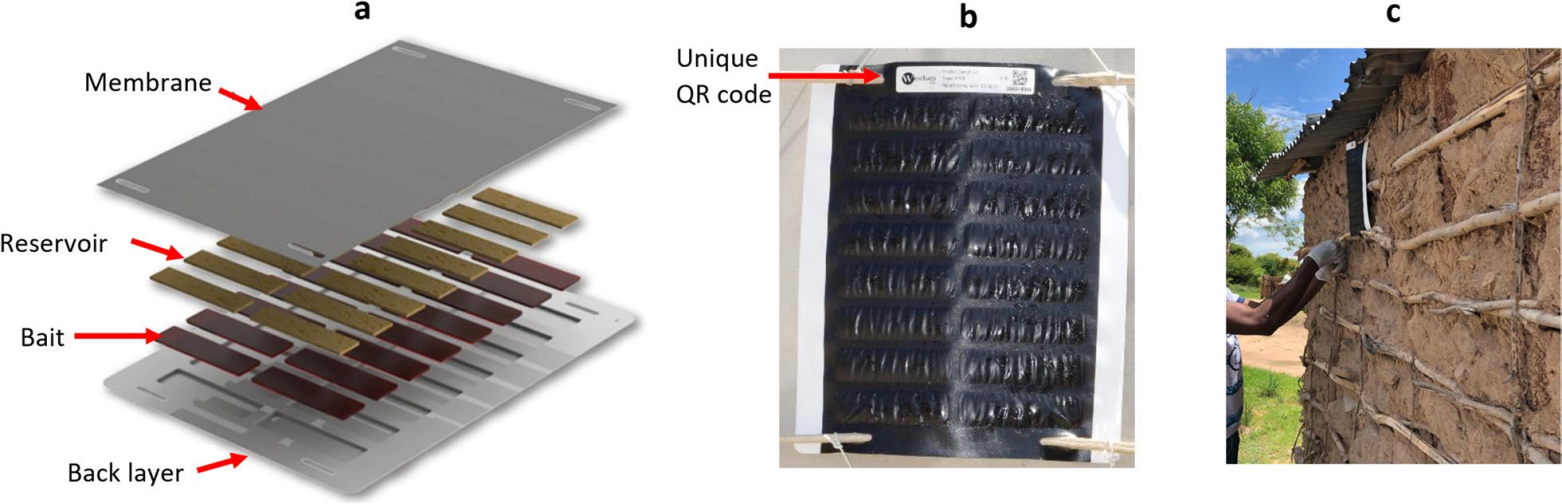
**a** an illustration of the Sarabi v1.2 ATSB station design, **b** shows the ATSB station with an attached unique QR code, and mounted on bamboo sticks, while **c** shows installation of an ATSB station on the outside wall of a household structure during the trial

In countries like Zambia where malaria is endemic, vector control measures such as the distribution of insecticide-treated nets (ITNs) and indoor residual spraying (IRS) form a core part of malaria control strategies [1, 4, 5]. Attractive targeted sugar baits (ATSBs) have been proposed as a potential new class of vector control measures to supplement existing tools in addressing growing insecticide resistance in mosquitoes [6, 7] as well as their outdoor biting behaviour [8, 9]. ATSBs take advantage of the natural sugar feeding behaviour of mosquitoes, which rely on sugar sources such as plant tissue and nectar from flowers for sustenance [10, 11]. By providing a bait containing sugar plus an ingestion toxicant (Fig. 1a), ATSBs attract and kill sugar-feeding mosquitoes. This ‘attract and kill’ mechanism has been demonstrated in some studies where mosquitoes have been shown to feed on the ATSBs [12–14] and that this intervention reduced mosquito density [12]. The suitability of ATSBs as a vector control tool will also depend on evidence of a public health impact on malaria (Ashton et al*.,* pers.commun.), as well as operational feasibility and cost effectiveness (Mancuso et al*.*, pers.commun.) at scale-up.

To generate evidence of public health impact, cluster-randomized control trials (cRCT) were conducted in Zambia, Kenya, and Mali to determine the efficacy of the Sarabi v1.2 ATSB station (Westham, Hod-Hasharon, Israel) in different settings, and in the context of existing vector control (ITNs or IRS) [11]. The main aim was to investigate the impact of these ATSB stations on malaria incidence in the context of universal ITN and/or IRS, with additional outcomes including impact on malaria prevalence, impact on the mosquito population and ATSB station durability, among others. The Sarabi v1.2 ATSB station contains date syrup (the sugar), dinotefuran (the toxin), and Bitrex (to deter human ingestion).

The first trial was conducted in western Zambia—a rural, high malaria transmission setting with ample natural vegetation [15], between 2021 and 2023. During the cRCT, 35 clusters each were randomly assigned to the control and intervention arms. The control arm received standard of care vector control, while the intervention arm received both the standard of care vector control and ATSB stations were deployed on eligible household structures. Year 1 of intervention deployment ran between November 2021 and June 2022, while year 2 ran from October 2022 to June 2023, during the transmission season of each year. Full details of the trial design, epidemiological results and entomological results are presented elsewhere [11, 16] and Ashton et al*.* (pers.commun.).

Critical to this efficacy trial was maintaining high coverage of ATSB stations in good condition during the two deployment seasons. This required a robust monitoring system to address damage to ATSB stations and fill any emerging coverage gaps. The ATSB station deployment and monitoring approaches for both years of the trial are detailed in Orange et al*.* [17]. Routine monitoring during both years identified tears and mold developing on the surface of ATSB stations as the main types of damage to ATSB stations in this setting. The prevalence of overall damage and different damage types, as well as the temporal and spatial trends in these factors have implications for operational feasibility, cost, and sustainability of ATSB station deployment as a vector control strategy. Additionally, understanding the drivers of damage to ATSB stations can inform future ATSB product development.

Karabo et al. [18] conducted a physical durability study assessing the median survival time of ATSB stations in a sub-set of trial clusters using a rolling cohort approach. In that study, survival failures occurred when installed ATSB stations went missing or met one or more pre-defined replacement criteria due to damage. The study was conducted on 1107 ATSB stations that were installed on structures within 10 purposively selected households in 20 of the 35 intervention clusters in year 2 of the trial. The study also explored how specific structure characteristics may have contributed to the survival of ATSB stations. A key determinant of survival was the level of protection from rainfall and sunshine afforded by the position where ATSB stations were installed on the structure (i.e. the size of the roof overhang and whether the ATSB station was able to be installed high on the wall under the roof overhang). The study found that ATSB stations that were well protected had a median survival time greater than 218 days, while those ATSB stations with no protection had median survival time of 90 days.

The current study provides a more comprehensive assessment of damage to ATSB stations in this setting by investigating: (1) temporal and spatial trends in the prevalence of overall damage and damage types across the entire study site; and (2) the characteristics of the structure and environment driving ATSB station damage.

## Methods

ATSB stations during both trial years were deployed during the malaria transmission season in Zambia, which coincided with the rainy season from mid-November to April, to maximize potential impact on malaria incidence. Definitions of variables related to ATSB station damage changed slightly between year 1 and year 2 of deployment. Consequently, this study focuses solely on the collection and analysis of data from year 2.

### Data collection

Data in this trial was collected using CommCare (Dimagi, Cambridge MA), a digital data collection application installed on Android mobile devices. Data collection for year 2 ran from 31 October 2022 to 30 June 2023, which is referred to in this study as the ‘deployment period’. This period included an ATSB installation campaign, ATSB monitoring and an ATSB removal campaign.

#### Installation campaign

During the installation campaign (31 October–12 November 2022), consent was obtained from households within the 35 intervention clusters. Two ATSB stations were installed on the outside walls of each eligible structure in the household by community based ATSB monitors who were recruited and trained to carry out this work. The unique QR code, installation date and GPS location of each ATSB station was recorded. Further details on eligibility criteria and implementation are reported elsewhere [17]. In what follows, eligible household structures will be referred to simply as ‘structures’.

#### Monitoring period

Between 1 December 2022 and 9 June 2023, ATSB stations were routinely monitored by ATSB monitors who visited every ATSB station at least once every 2 months to collect information on its physical condition, and replace it if it met pre-defined replacement criteria due to damage. Additional visits were conducted to address damage reported by households, to fill in any coverage gaps arising from new structures that had been built during the monitoring period, or replace ATSB stations that were missing or removed for reasons other than damage.

**Fig. 2:**
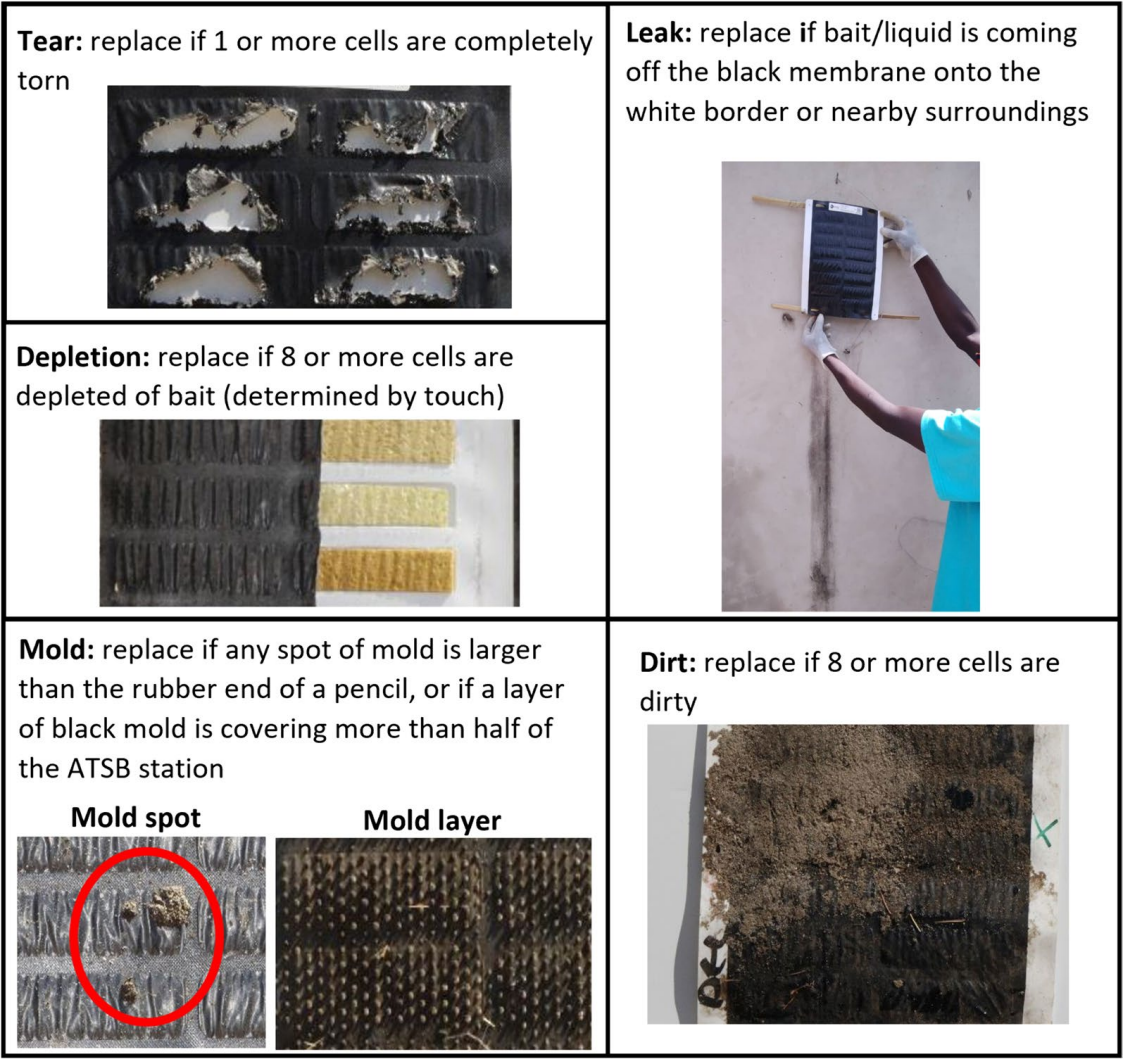
Images showing different ATSB station damage types, and their respective replacement criteria due to damage

Replacement criteria for damaged ATSB stations were defined by the ATSB Trial Partners group considering the need to maintain attractancy of ATSB stations to target vectors, sustain community acceptance, and mitigate potential adverse impact to humans and the environment. The criteria were defined for five damage types including: surface tears; mold growth; bait leakage; depletion (drying out) of the bait; and accumulation of excessive dirt on the surface of the ATSB station (Fig. 2).

During monitoring visits to ATSB stations, ATSB monitors recorded information on all five damage types, specifying if replacement criteria were met for each damage type. Once an ATSB station was damaged as per Fig. 2, it was removed and replaced, with the replacement’s QR code recorded and linked to the damaged ATSB station in the data. Further details on the replacement criteria and procedures are reported elsewhere [17]. In what follows, ATSB stations meeting any replacement criteria due to damage will be referred to as ‘damaged ATSB stations’.

Where ATSB stations had been removed for any reason other than damage, and could be recovered by ATSB monitors, they were recorded and withdrawn from the trial. Withdrawals typically occurred when structures collapsed, consent was withdrawn by households, or if ATSB stations were found discarded in the community. Where ATSB stations could not be recovered, they were left unaccounted for at the end of the trial.

Additional ATSB station monitoring was conducted for the ATSB physical durability study described in Karabo et al*.* [18]. The small number of ATSB stations recruited into the physical durability study underwent additional monitoring by specially trained data collectors conducting monthly scheduled visits, recording information on ATSB station condition and structure characteristics. Damaged ATSB stations were removed and replaced using the same protocol as the routine monitoring.

Towards the end of the routine monitoring period (between 1 and 9 June 2023), ATSB monitors prioritized responding to damaged ATSB stations over visiting non-damaged ATSB stations or installing ATSB stations on new structures as monitoring activities wound down. During this wind-down period, damaged ATSB stations continued to be replaced per protocol.

The questionnaire used during data collection for routine monitoring is included in the supplementary materials (Appendix 1).

#### Removal campaign

Between 15 and 30 June 2023, all ATSB stations were removed from structures. Data was collected on date and location of removal, and condition of the ATSB station. Additional information was collected on structure characteristics including how protected from rainfall and sunshine ATSB stations on the structure had been, and the structure’s wall and roof material (See Appendix 2 for questionnaire).

### Analysis

All data cleaning and analyses were conducted in the R programming language, version 4.3.1 [19]. Analysis includes data collected during the installation campaign, both routine and durability monitoring, and the removal campaign.

#### ATSB-level analysis

The ATSB-level analysis is descriptive, focusing on the prevalence of overall ATSB station damage and damage types among all ATSB stations throughout the deployment period, as well as how this changed over time, and differed between clusters. We investigate ATSB stations’ daily damage proportion—defined as the proportion of monitoring visits each day where a damaged ATSB station was observed. This outcome gives an indication of the intensity of ATSB station replacement due to damage during the monitoring period. Temporal analyses are restricted to the monitoring period from 1 December 2022 to 31 May 2023, excluding the wind-down monitoring period (1–9 June 2023).

#### Structure-level analysis

The structure-level analysis explores the prevalence and rate of damaged ATSB stations on structures throughout the deployment period, and how this is distributed in space across the trial site. We also investigate the distribution of different damage types across the trial site through spatial analysis, as well as the structure characteristics and environmental drivers of damage to ATSB stations through regression analysis.

Throughout the deployment period, ATSB stations were tracked through their unique QR code (Fig. 1b). To generate structure-level outcomes, a profile was constructed for each structure specifying all the ATSB stations that had been installed and whether they had been damaged during deployment. This was done by linking the QR codes of the ATSB stations through data collected at installation, removal, and where relevant, during replacement of damaged ATSB stations.

For structures included in this analysis, the structure profile begins during the installation campaign. Each structure profile consists of two ‘positions’ corresponding to the first and the second ATSB stations installed at the first structure visit. The structure profile ends at the removal campaign when all ATSB stations were removed from structures. Structures that became eligible only during the monitoring period, or where ATSBs were withdrawn or lost to follow-up were excluded. This approach ensured that the analysis only included structures participating in the full length of the deployment period, and that the damage status of all ATSB stations that had been installed on the structure could be ascertained. Supplementary Fig. S1 illustrates construction of the structure profile, while supplementary Fig. S2 presents the resulting sample size.

Two mixed effects generalized linear models (GLMs) were used to investigate the drivers of damage to ATSB stations at structure-level. The first model (Model 1) investigates the odds of a structure having at least one damaged ATSB station in its profile at any time. The outcome variable is binary, thus Model 1 is a logistic regression with a logit link function. The second model (Model 2) investigates the rate of damage to ATSB stations on structures by considering the number of excess ATSB stations in the structure profile. Excess ATSB stations are defined as those exceeding 2 ATSB stations per structure (i.e. number of replacements made). Thus, the outcome variable for Model 2 is discrete, and the model is a negative binomial regression. Both models contain structure-level fixed effects and a random effect on cluster. Model 1 was implemented using the stats package, while Model 2 was implemented using the MASS package [20] in R.

The covariates considered in both models were selected based on findings from the physical durability study (Karabo et al. [18]), observations by the ATSB monitoring teams and descriptive results from in the ATSB-level analysis. The covariates selected included the level of protection from rain and sunshine for ATSB stations that had been in positions 1 and 2 in the structure profile, wall and roof material of the structure, day-time and night-time land surface temperature (LST), enhanced vegetation index (EVI), land cover, and rainfall.

The protection level of ATSB position 1 and 2 of the structure profile was classified descriptively as: ‘well protected’, ‘somewhat protected’, ‘little or no protection’ based on the extent to which the ATSB station was tucked under a roof overhang and protected from direct sunlight and rain, as well as how far above the ground the ATSB station had been installed. These data were recorded during the removal campaign, together with description of roof and wall materials of structures. Protection level was aggregated to the structure. Where the protection level was discrepant between position 1 and 2 on the structure, this was re-classified to the lower class, and where the protection level for one of the positions was missing, we retained protection level of the known position (supplementary Table S1).

Day-time and night-time LST, were obtained from the National Aeronautics and Space Administration’s (NASA’s) Moderate Resolution Imaging Spectroradiometer (MODIS) as daily rasters at a spatial resolution of 1 km, EVI data were obtained from MODIS as 16-day rasters at a spatial resolution of 250 m, while monthly rainfall data were obtained from Climate Hazards Group InfraRed Precipitation with Station data (CHIRPS) v2.0 as rasters at a spatial resolution of 5 km [21]. Day-time and night-time LST, EVI and rainfall were obtained for October 2022–June 2023, aggregated to averages over this time-period, and standardized to aid in interpretation of their relative importance.

Land cover data from 2021 was obtained from MODIS as a raster with a spatial resolution of 250 m, and the categories assigned for structures in this analysis included: ‘croplands’, ‘grasslands’, ‘savannas’, ‘urban and built-up lands’, and ‘woody savannas’. All MODIS data were obtained using the MODIStsp package in R [22].

## Results

**Fig. 3:**
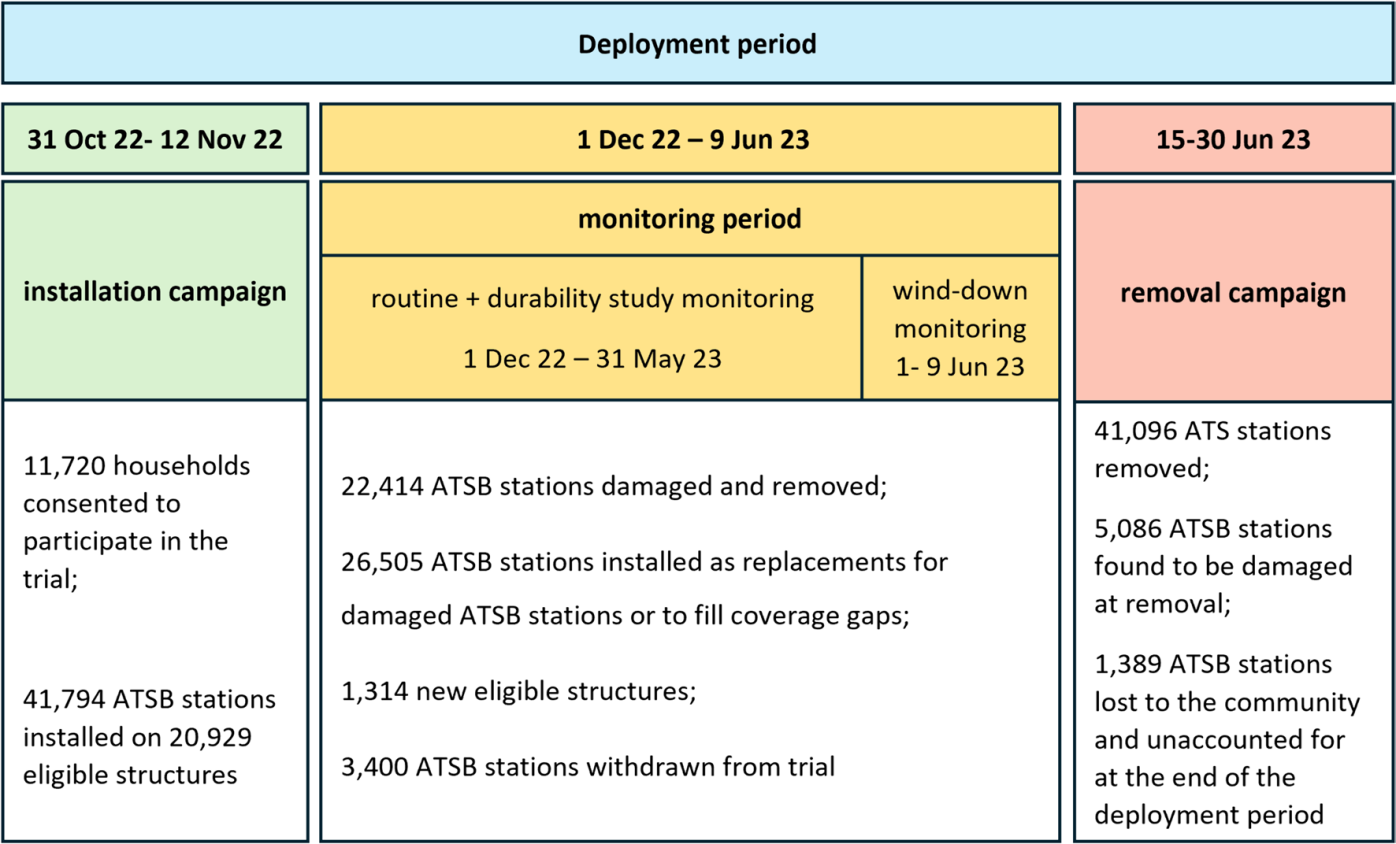
Timeline and overview of the ATSB deployment period for year 2 of the trial

The installation of 68,299 ATSB stations on 22,243 eligible structures was recorded during both the installation campaign and the monitoring period (Fig. 3). This represents 98.3% of all ATSB stations distributed to ATSB station monitors for installation. The 1.7% shortfall was attributable to wastage, as well as data entry and technical errors in CommCare during data collection.

The number of eligible structures and total ATSB stations both varied widely across the intervention clusters (360–1193 eligible structures and 1012–3512 ATSB stations per cluster, supplementary Fig. S3). The number of installations per cluster was influenced by both the number of eligible structures, as well as the number of damaged ATSB stations that needed replacement in the cluster.

### ATSB-level results

**Fig. 4:**
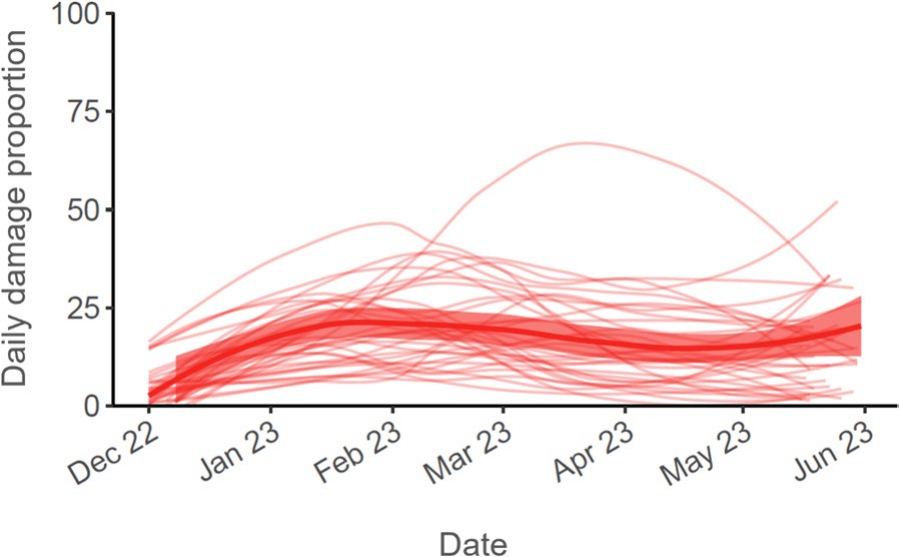
Proportion of monitoring visits where ATSB stations are observed to be damaged, over time. The thin curves represent proportions for the 35 individual clusters, while the single bold curve represents overall proportions across all clusters. The curves are generated using LOESS, and the band around the overall curve represents 95% CIs. Data presented excludes wind-down monitoring

A total of 27,500 ATSB stations were observed to be damaged during the year 2 deployment period. The average daily damage proportion across all clusters was 16.1%, meaning that on average, nearly one in six monitoring visits to ATSB stations per day ended in replacement of damaged ATSB stations. Figure 4 shows how daily damage proportion evolved over time, with a peak across all clusters occurring between January and February, and then again in May. Between clusters, daily damage proportion over time also varied widely, with one cluster having a particularly large peak in March.

**Fig. 5:**
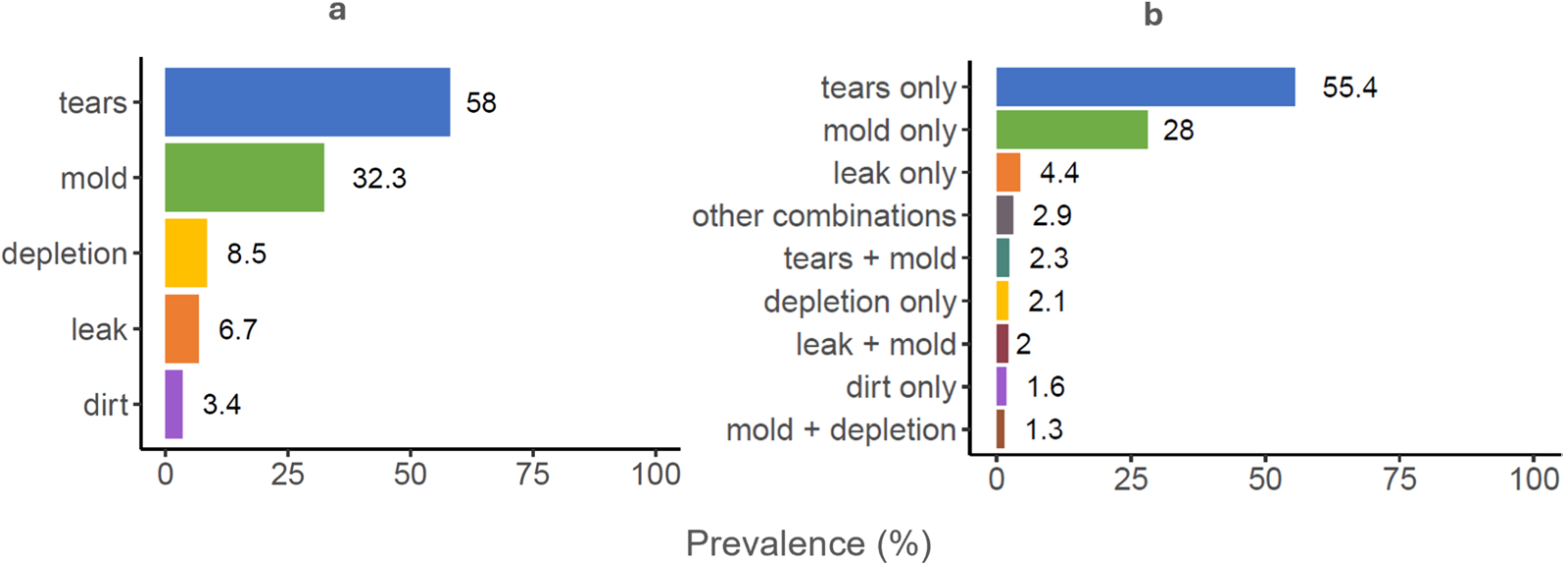
Prevalence of the different damage types among damaged ATSB stations throughout the year 2 deployment period. **a** shows the overall prevalence of the specified damage types, while **b** shows the prevalence of specific combinations of damage

Among damaged ATSB stations, the most prevalent damage types observed in this analysis were tears (58.0%) and mold (32.3%), while depletion, leaks, and dirt were less common (8.5, 6.7, 3.4%, respectively [Fig. 5a]). The majority of damaged ATSB stations had only one damage type. Only 8.5% of ATSB stations had more than one damage type (Fig. 5b), most commonly ‘tears plus mold’ (2.3%), and ‘leak plus mold’ (2.0%).

**Fig. 6:**
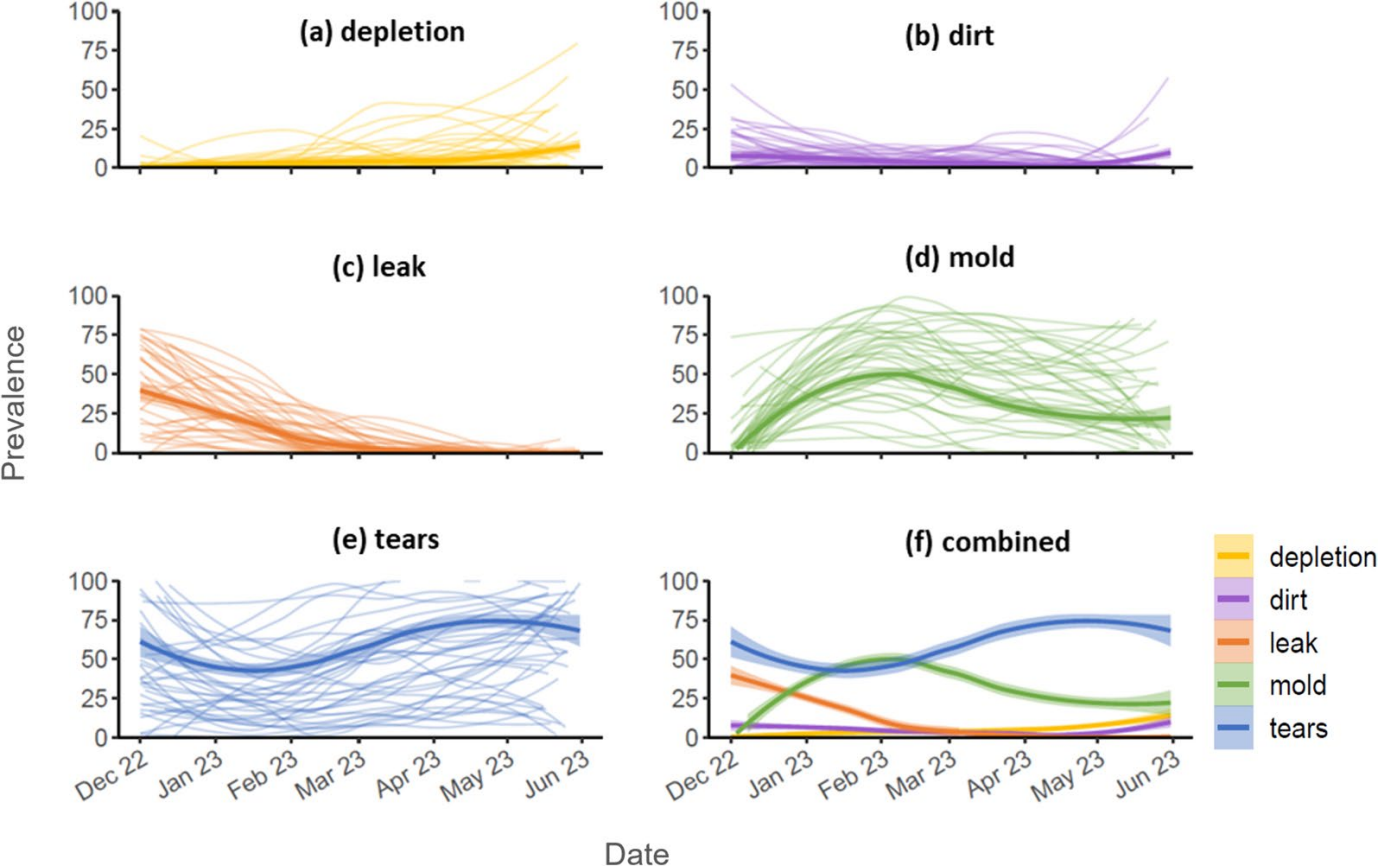
Prevalence of the different damage types among damaged ATSB stations, over time. In (**a**–**e**), The thin curves represent prevalence for the 35 individual clusters, while the single bold curve represents overall prevalence across all clusters. **f** shows the overall curves in (**a**–**e**) together over time. The curves are generated using LOESS, and the band around the overall curves represents 95% CIs. Data presented excludes wind-down monitoring

Among damaged ATSB stations, temporal trends in the prevalence of different damage types varied substantially depending on the damage type (Fig. 6). Leaks were more prevalent at the start of the monitoring period and decreased by March (Fig. 6c). The prevalence of mold damage peaked in February then remained high in some clusters, while decreasing in others (Fig. 6d). The prevalence of tears was consistently high (above 50.0%) throughout much of the monitoring period, with substantial variation between clusters (Fig. 6e).

There was an inverse temporal relationship between the prevalence of mold versus tears, with high prevalence of one corresponding to low prevalence of the other (Fig. 6f). This phenomenon occurred across all clusters (supplementary Fig. S4).

### Structure-level results

Of the 22,243 structures on which ATSB stations were installed, 18,890 (84.9%) qualified to be included in this structure-level analysis.

For most of these structures (58.4%), ATSB stations were installed in well protected positions. The most common wall materials were ‘wood with mud’ (46.5%) and’mud brick’ (32.4%). Roof material was predominantly ‘thatch’ (69.0%) with ‘iron sheets’ making up 28.7%. A large proportion of structures with a ‘thatch’ roof (77.7%) had well protected ATSB stations compared to 15.6% of structures with an ‘iron sheet' roof. Structures were most commonly located in ‘grasslands’ (43.3%), with substantial proportions located in ‘savannas’ (32.6%) and ‘woody savannas’ (20.8%). Further breakdown of these structure characteristics, as well as images of the most common structures at the study site are provided in the supplementary materials (Figs S5 and S6).

Of the structures included in this analysis, 8622 (45.6%) had at least one damaged ATSB station in their structure profile. The prevalence of structures with at least one damaged ATSB station varied by cluster, ranging from 23.3 to 85.4% (supplementary Fig. S7).

The prevalence of different damage types at structure-level echoed observations in the ATSB-level analysis where tears and mold were the most prevalent damage types (Table 1). Interestingly, while in the ATSB-level analysis tears were more common than mold, in the structure-level analysis, this is reversed. This suggests that damage due to tears led to a higher turnover of ATSB stations on specific structures, while damage due to mold was spread out over more structures. This trend is further supported by supplementary Table S2, which indicates that 83.1% of structures with high ATSB station turnover (more than 4 damaged ATSB stations) had all affected ATSB stations showing damage from tears, in contrast to only 0.4% for mold damage.

**Table 1:** Prevalence of different damage types observed on the 8,622 structures with at least one damaged ATSB station

Damage type	Structures where a damaged ATSB station exhibited the specified damage type, n (% of structures with at least one damaged ATSB station)
Tears	3927 (45.6%)
Leak	1011 (11.7%)
Mold	4906 (56.9%)
Depletion	819 (9.5%)
Dirt	546 (6.3%)

**Fig. 7:**
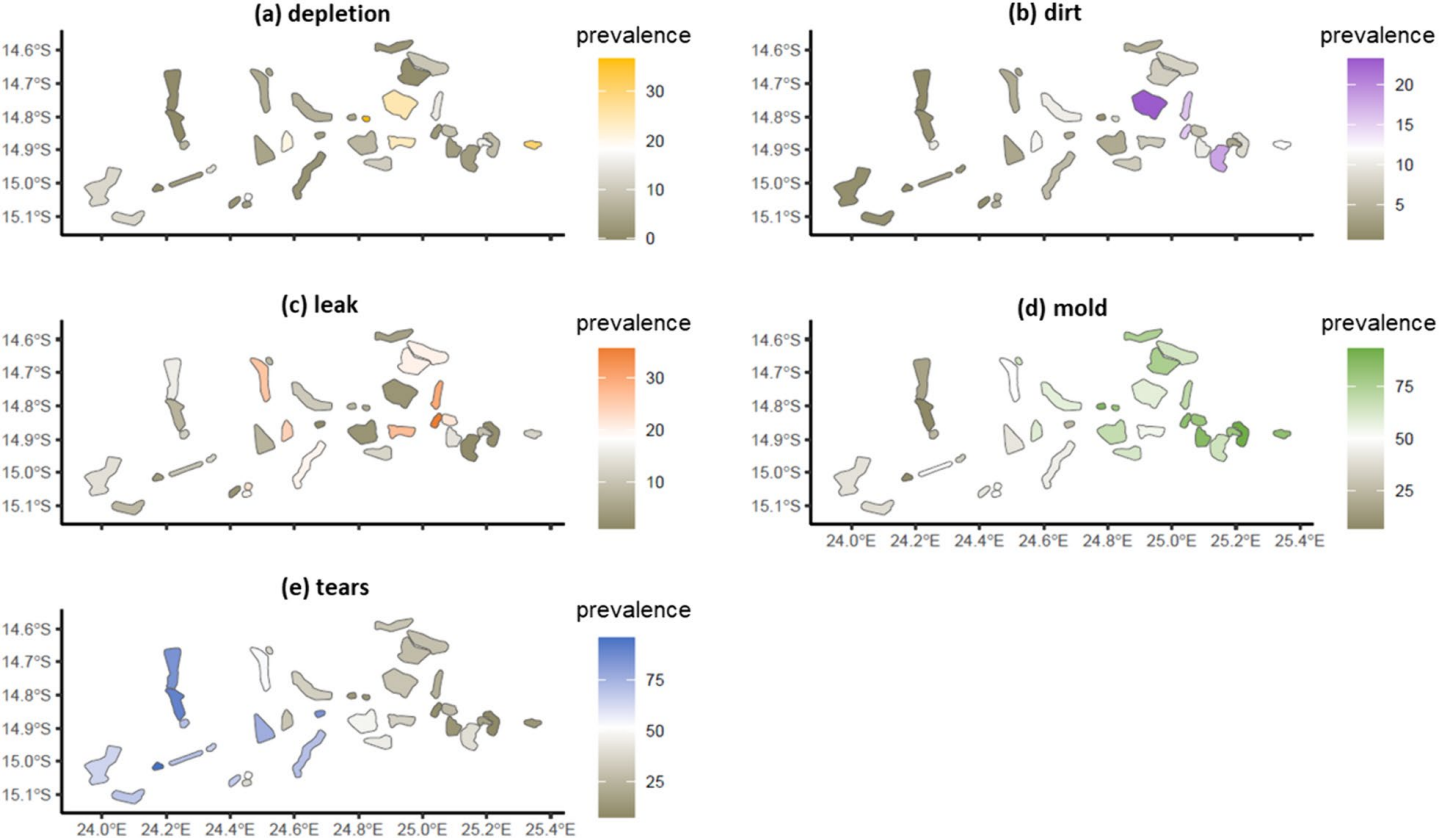
Prevalence of different damage types among structures with at least one damaged ATSB station across the intervention clusters

Spatial trends in the prevalence of different damage types were observed across intervention clusters. Depletion and dirt and mold were more common in the east of the trial site, while tears were more prevalent in the west (Fig. 7). Like the temporal relationship observed between tears and mold in Fig. 6f, an inverse spatial relationship between tears and mold was observed in the structure-level analysis (Fig. 7).

**Fig. 8:**
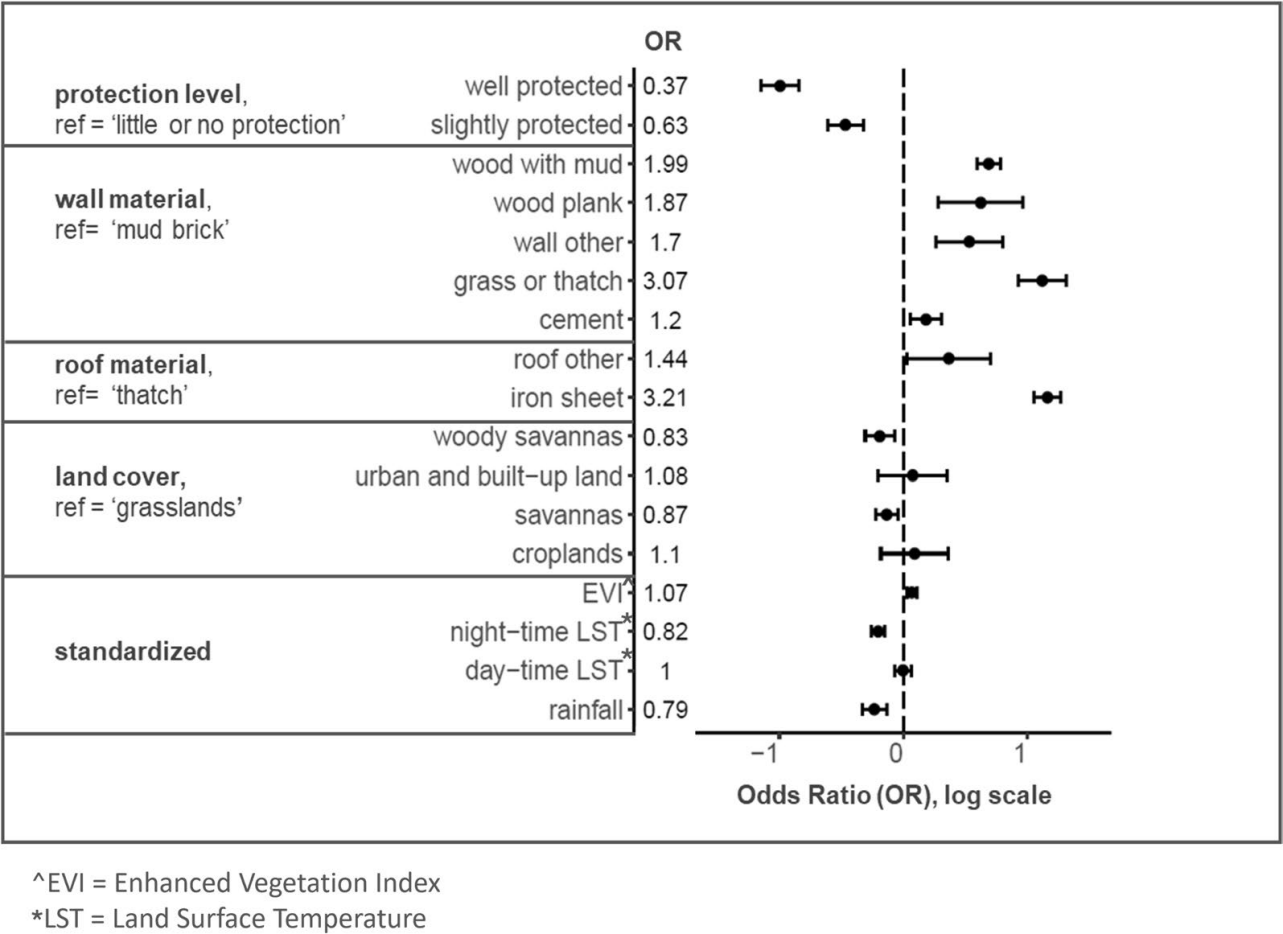
Model 1 outputs indicating the odds of a structure having at least one damaged ATSB station in its structure profile. Odds Ratio (OR) values are indicated to the right of the covariate names and on the plot, these are presented on the log scale along with their 95% CIs. Negative values on the log scale indicate decreased odds while positive values indicate increased odds of at least one damaged ATSB station in the structure profile

Results from the regression Model 1 indicate that both structure characteristics and environmental factors significantly impacted the odds of a structure having at least one damaged ATSB station (Fig. 8). The odds of damage reduced significantly from structures with ‘little to no protection’ to ‘well protected’. The odds of damage were significantly increased for all wall types in comparison to ‘mud brick’, and significantly increased with an ‘iron sheet’ roof compared to a ‘thatch’ roof. Land cover was significantly protective if the structure was located in ‘woody savannas’ or ‘savannas’ compared to ‘grasslands’. An increase of one standard deviation in EVI significantly increased the odds of at least one damaged ATSB station on structures, while increases of one standard deviation in night-time LST and rainfall were both significantly protective.

**Fig. 9:**
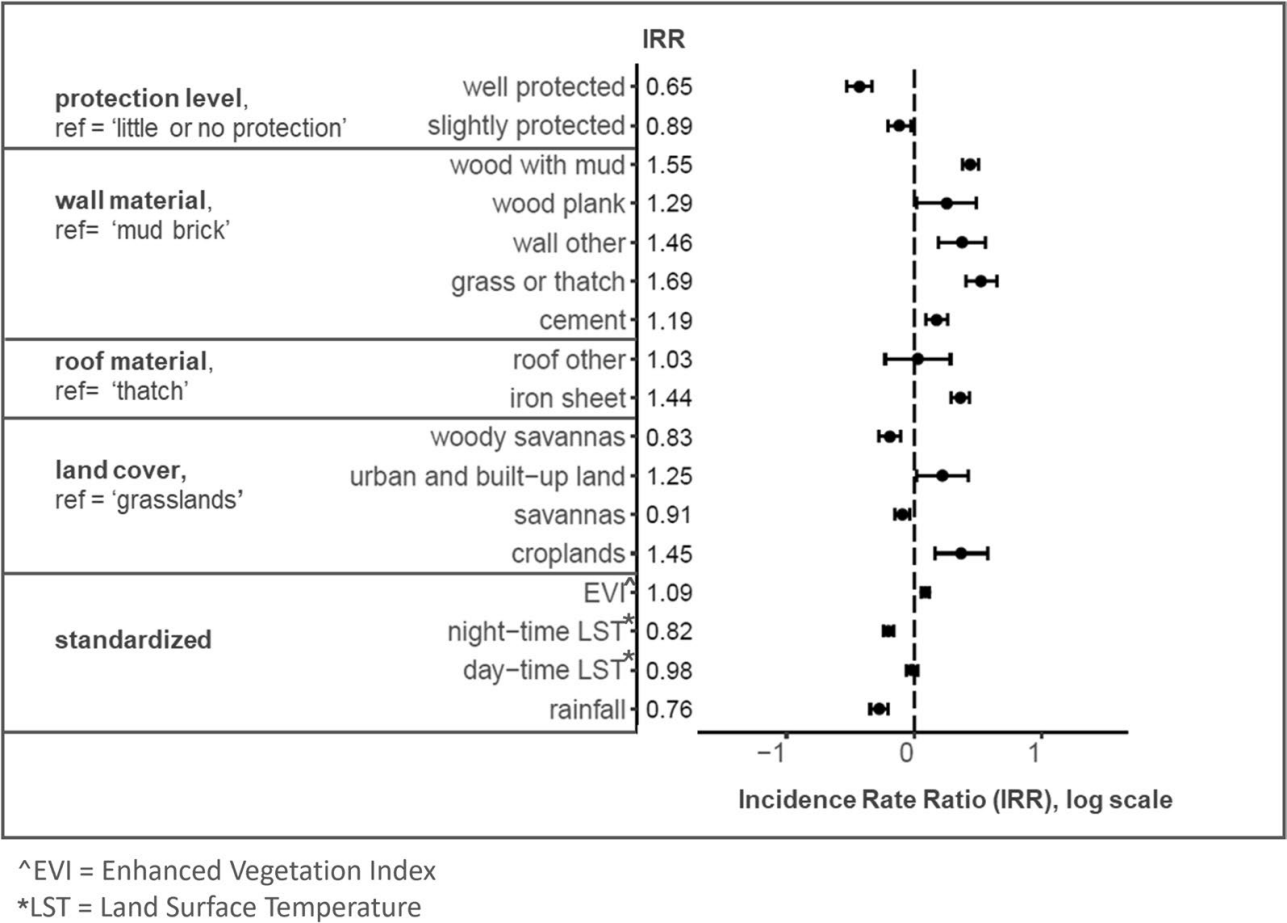
Model 2 outputs indicating the rate of damaged ATSB stations on structures. Incidence Rate Ratio (IRR) values are indicated to the right of the covariate names and on the plot, these are presented on the log scale along with their 95% CIs. Negative values on the log scale indicate decreased rate while positive values indicate increased rate of damaged ATSB station in the structure profile

Model 2 regression results, which indicate the impact of covariates on the rate of damage to ATSB stations on structures, echoed Model 1 results in the direction and significance of effect for the different covariates in all but two cases (Fig. 9). For land cover, ‘urban and built-up land’ and ‘croplands’ both significantly increased the rate of damage to ATSB stations on structures compared to ‘grasslands’ but were found in Model 1 to have no impact on the odds of a structure having at least one damaged ATSB station.

## Discussion

This analysis of ATSB monitoring data from year 2 of the Zambia ATSB trial was undertaken to examine temporal and spatial trends in ATSB station damage and identify factors associated with this damage. The prevalence and distribution of overall damage as well as different damage types was heterogeneous, with temporal and spatial trends identified. On average, nearly one in six monitoring visits to ATSB stations per day ended in replacement of damaged ATSB stations, and tears and mold were the most prevalent damage types. Among damaged ATSB stations observed per day during monitoring, the prevalence of tears maintained above 50.0% through most of the monitoring period, while the prevalence of mold increased steadily during the first few months, peaking in February. Over the deployment period, 45.6% of structures had at least one damaged ATSB station, however this varied between clusters. Significant drivers of damage to ATSB stations included ATSB stations’ protection level from rainfall and sunshine; roof and wall material of the structure; night-time temperature; rainfall; EVI, and land cover.

The mechanisms of damage to ATSB stations were not directly studied during the trial, and further investigations are required to specify these mechanisms. However, results from this study provide some insights into possible environmental and structure-level drivers of damage.

Results from the study indicate that damage due to tears, believed to be largely caused by rodents, was consistent over time but spatially heterogenous, suggesting that factors related to rodent habitat and availability of food, such as EVI and land cover [23], may be important predictors of tears. The high turnover of ATSB stations due to tears suggests that modification to the ATSB product design may be necessary to discourage rodents from feeding on ATSB stations in similar settings.

Factors such as long-term relative humidity, temperature and wetness duration on the surface contribute to mold growth [24]. Therefore, protection level, temperature, wall material, roof material and rainfall may all contribute to the mechanisms behind this damage type. Different roof and wall materials may affect heat and moisture retention, while night-time temperature may impact condensation levels. Similar to mold growth, depletion is likely influenced by the ambient temperature and moisture surrounding the ATSB station over time.

Leaks were observed at the start of the deployment period in Zambia during the rainy season from mid-November to April [5]. Structures with iron roofs tended to have short overhangs, and therefore less protection for ATSB stations from rainfall compared to thatched roofs that typically had larger overhangs. Spatial variation in damage due to leaks, and mold, is likely attributable to differences in household construction between clusters. Given the significance of these two damage types, future ATSB product development should consider modifications to minimize them.

Temporal and spatial trends show that different damage types emerged at different times and were not evenly distributed across the trial site. ATSB stations were replaced as soon as any damage type was observed, leading to a higher representation of damage types that emerged earlier or at higher rates. Therefore, the different damage types can be considered competing risks. The prevalence of damage due to tears versus mold showed an inverse relationship, indicating that ATSB stations quickly replaced due to tears may not have been deployed long enough to develop mold damage. Therefore, clusters which did not report high mold damage may simply have had an ATSB station turnover too high to experience mold damage.

In this trial ATSB stations were deployed during the malaria transmission season to maximize the potential impact on malaria prevalence and incidence. Understanding temporal trends in damage prevalence could help with operational strategies, such as anticipating periods of high damage when larger stocks of ATSB stations may be required, or the optimal timing of monitoring visits during deployment after a specific damage process is anticipated to have ended. Information on structure characteristics at a deployment site may also inform expected damage types, and when damage is likely to occur.

Utilizing geostatistical methods for spatial analysis could help predict areas where ATSB stations are less likely to be damaged, or where the rate of damage to ATSB stations is likely to be low. This information can help target deployment of ATSB stations and optimize monitoring and replacement strategies, ultimately improving operational feasibility and cost-effectiveness.

The definition of damage to ATSB stations needs to be standardized for future use [17]. The replacement criteria for damage, which were set in the context of a cRCT, may have been overly conservative and require further evidence-based refinement. Understanding how different types and levels of damage impact the attractiveness and bio-efficacy of ATSB stations could help determine when these factors render an ATSB station ineffective, and therefore guide standardization of replacement criteria.

In year 2 of the study, ATSB stations were widely accepted by the communities [25]. However, the impact of ATSB station damage on acceptability of ATSB stations by community members requires consideration. Similar to ITNs, poor physical condition of the ATSB stations may impact the use of this intervention [26]. Future ATSB deployments may consider that perceptions about ATSB stations attracting rodents may become problematic in areas with sustained rodent damage. Additionally, leaks- which often stain the wall on which the ATSB station is installed- as well as mold damage may be unsightly to households and contribute to rejection of ATSB stations by households.

Lastly, the prevalence and rate of ATSB station damage may be different for the Mali and Kenya trials, which were conducted in different settings compared to Zambia. Differences in household structure construction, as well as differences in macro-level environmental factors may reveal a different or wider range of factors that influence the likelihood and rate of damage to ATSB stations in different settings.

## Conclusion

Damage to ATSB stations was common and wide-spread during the 7-month deployment period, was temporally varied across this period, and was spatially varied across the trial site. Significant drivers of damage to ATSB stations included household structure characteristics as well as environmental factors. These findings offer some indication of factors associated with damage to ATSB stations and the trends in damage that can be anticipated in similar settings. If the infection and disease burden reduction efficacy of ATSBs merits their public health deployment, further investigations are merited to evaluate the mechanisms of damage in order to develop strategies to minimize this damage, and to determine the impact of different damage types on the attractiveness and bio-efficacy of ATSB stations.

### Supplementary Information

Additional file 1

## Data Availability

De-identified data are available from the last author (rashton@tulane.edu) on reasonable request. Following publication of forthcoming secondary analyses of trial data, the de-identified trial dataset will be posted on a public repository.

## Abbreviations

ATSB: Attractive targeted sugar bait
CHIRPS: Climate Hazards Group InfraRed Precipitation with Station data
cRCT: Cluster-Randomized Control Trial
EVI: Enhanced vegetation index
GLM: Generalized linear model
IRS: Indoor residual spraying
ITNs: Insecticide-treated nets
LST: Land surface temperature
MODIS: Moderate resolution imaging spectroradiometer
NASA: National Aeronautics and Space Administration
