# Supplementary material for: Evaluating trends in damage to attractive targeted sugar baits (ATSBs) deployed during the second year of a two-year Phase III trial in Western Zambia
Source: Malar J. 2024 Aug 29;23:263. doi: 10.1186/s12936-024-05089-5 (PMC11363357; doi:10.1186/s12936-024-05089-5)
Supplement: Supplementary file 1 — Additional file 1 [file 12936_2024_5089_MOESM1_ESM.docx]

# Supplementary materials

**“Evaluating trends in damage to Attractive Targeted Sugar Baits deployed during the second year of a two-year Phase III trial in Western Zambia.**

Irene Kyomuhangi, Joshua Yukich, Kochelani Saili, Erica Orange, Mundia Masuzyo, Mwansa Mwenya, Patricia Mambo, Busiku Hamainza, Joe Wagman, John Miller, Javan Chanda, Kafula Silumbe, Megan Littrell, Thomas P. Eisele, Ruth A. Ashton

**Figure S1:** This figure describes one scenario where ATSB stations on the structure were damaged and replaced during the monitoring period. Different structures had different profiles depending on whether ATSB stations had been damaged, had been withdrawn, or were unaccounted for at the end of the trial.


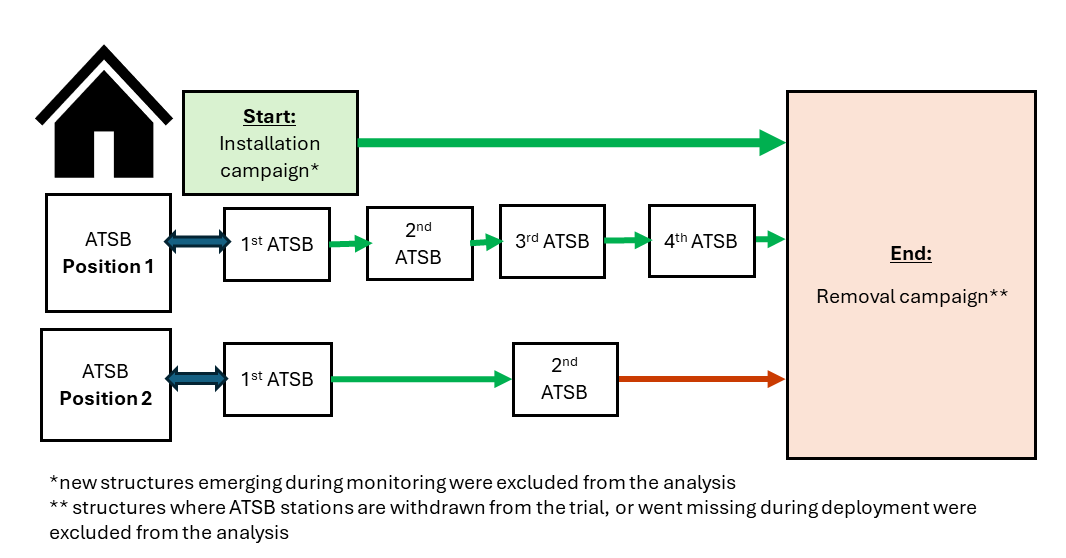


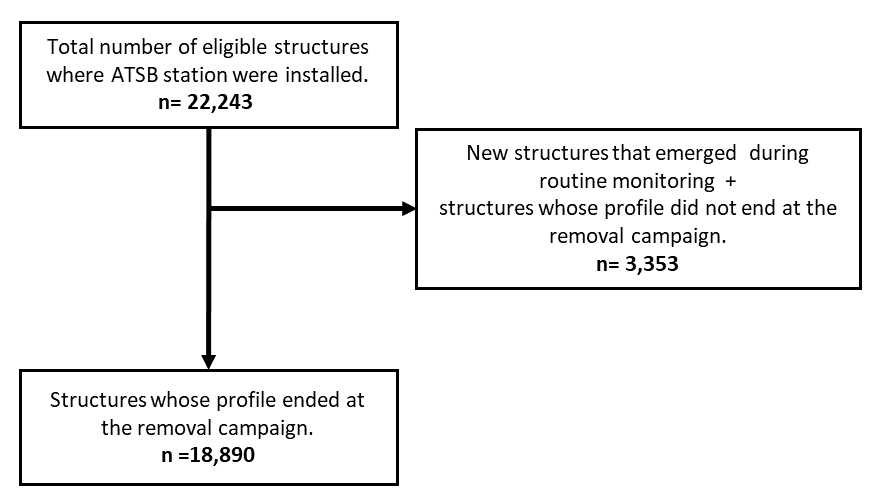
 **Figure S2:** Sample size for the structure-level analysis.

**Figure S3:** (a) shows the number and spatial distribution of eligible structures across the intervention clusters, while (b) shows the number and spatial distribution of ATSBs ever installed in these clusters

*
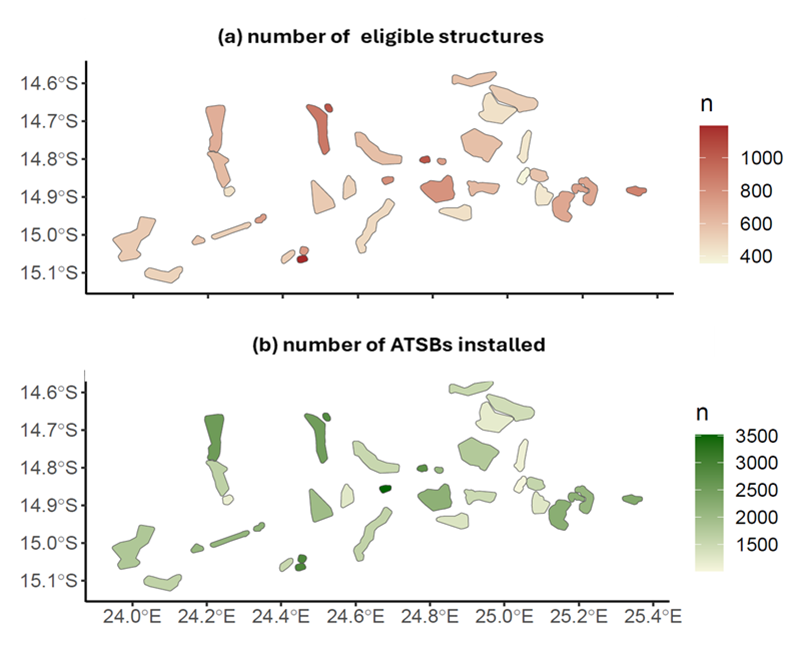
*

**Table S1**: Re-classification of protection level for ATSB stations on structures. The diagonal **bold** numbers in black represent structures where protection level for ATSB position 1 and 2 are concordant. The numbers in red represent structures where protection level is not concordant. The colour fill of each cell represents the final classification of protection-level for the structure. Cells with a **green fill** have a final classification of ‘well protected’, cells with a **blue fill** have a final classification of ‘slightly protected’, cells with an **orange fill** have a final classification of ‘little or no protection’, while cells with a **yellow fill** are ‘missing’.

|  |  | **ATSB position 2** | | | |
| --- | --- | --- | --- | --- | --- |
|  |  | well protected | slightly protected | little or no protection | missing |
| **ATSB position 1** | well protected | **10,079** | 720 | 279 | 531* |
|  | slightly protected | 829 | **2,883** | 401 | 253* |
|  | little or no protection | 225 | 375 | **1,121** | 137* |
|  | missing | 430* | 230* | 140* | **257** |

*74.5 % of pairs with protection-level data are concordant, hence the decision to assume ATSBs on positions with unknown protection level match their counterpart.


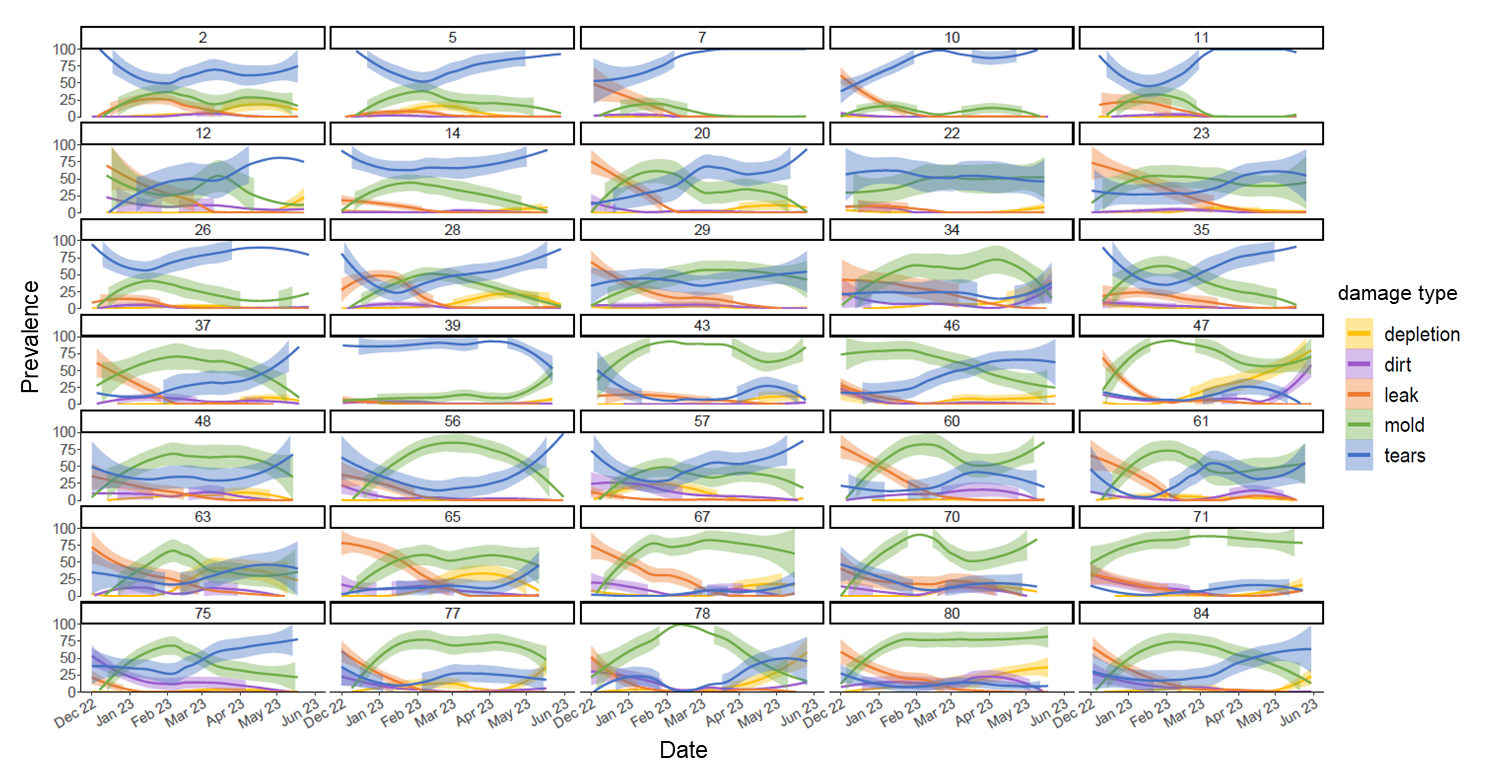
**Figure S4:** Prevalence of the different damage types among damaged ATSB stations in each intervention cluster over time. The numbers in the heading for each graph is the site number. The curves are generated using LOESS, and the band around the overall curves represents 95%CIs.

**Figure S5:** Structure characteristics of eligible structures in Year 2. Numbers represent the proportion (%) of the total.


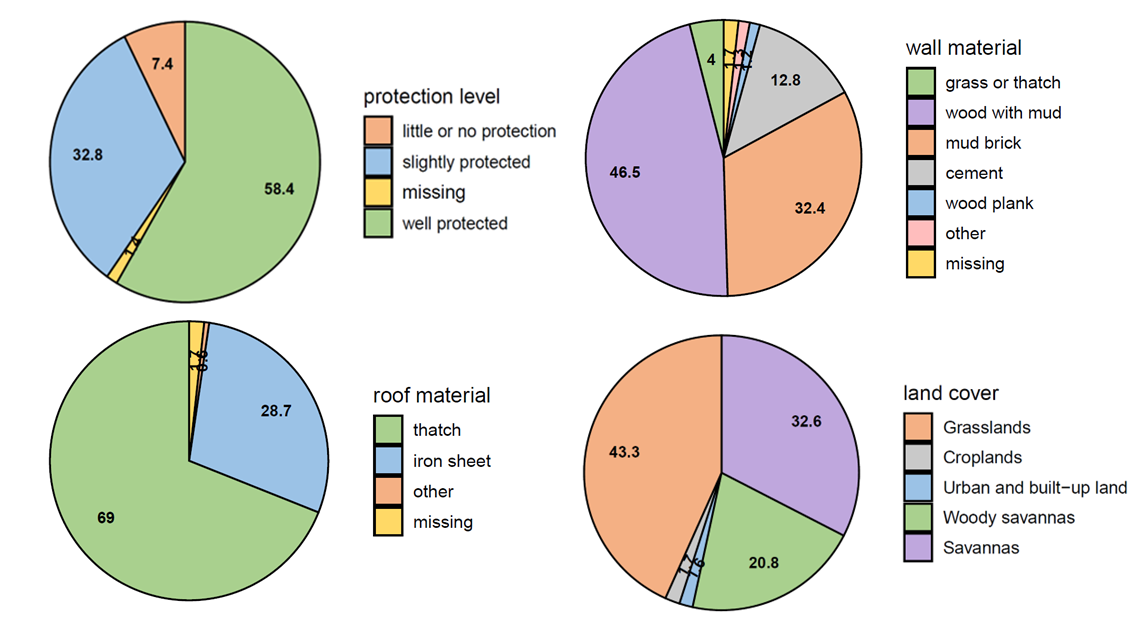


**Figure S6:** Examples of common structure types at the trial site (Arnzen *et al.*2024 [15])
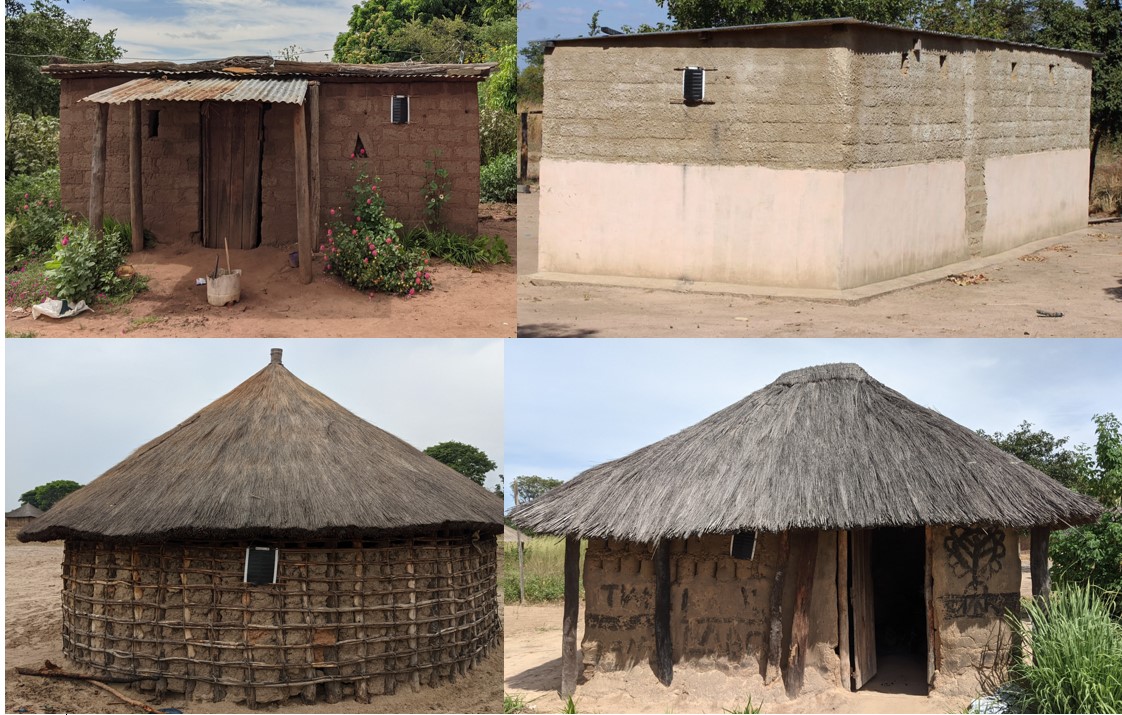


**Figure S7:** Prevalence of structures with at least 1 damaged ATSB station across the intervention clusters

*
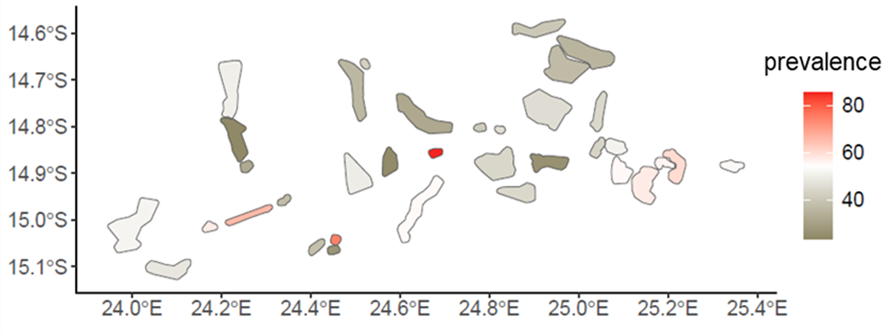
*

**Table S2**: Repeat damage types observed on the 8,622 structures with at least one damaged ATSB station in their structure profile.

|  | Damage type(s) observed on damaged ATSBs in the structure profile | | | |
| --- | --- | --- | --- | --- |
| Number of damaged ATSB stations in the structure profile  n = number of structures | All damaged ATSB stations had damage due to **tears**  n = number of structures  (Row %) | All damaged ATSB stations had damage due to **mold**  n = number of structures  (Row %) | All damaged ATSB stations had another consistent damage type  n = number of structures  (Row %) | Damaged ATSB stations did not have a consistent damage type  n = number of structures  (Row %) |
| **1**  n = 2,900 | - | - | - | - |
| **2**  n = 2,711 | n = 543  (20.0%) | n = 1279  (47.2%) | n = 197  (7.3%) | n = 692  (25.5%) |
| **3**  n = 1,056 | n = 371  (35.1%) | n = 189  (17.9%) | n = 24  (2.3%) | n = 472  (44.7%) |
| **4**  n = 729 | n = 477  (65.4%) | n = 31  (4.3%) | n = 5  (0.7%) | n = 216  (29.6%) |
| **> 4**  n =1,226 | n = 1,019  (83.1%) | n = 5  (0.4%) | n = 4  (0.3%) | n = 198  (16.2%) |

**Table S3:** regression outputs for Model 1 and Model 2. Significant codes are as follows: *** indicates p < 0; ** indicates p < 0.001; while * indicates p < 0.05

| **Covariate** | **Model 1**  **OR (95% CIs)** | **Model 2**  **IRR (95% CIs)** |
| --- | --- | --- |
| Protection level, ref = ‘little or no protection’ |  |  |
| slightly protected | 0.63 (0.54, 0.72)*** | 0.89 (0.81, 0.98)* |
| well protected | 0.37 (0.32, 0.43)*** | 0.65 (0.59, 0.72)*** |
| Wall material, ref = ‘mud brick’ |  |  |
| cement | 1.20(1.06, 1.36)** | 1.19 (1.09, 1.3)*** |
| grass or thatch | 3.07 (2.54, 3.71)*** | 1.69 (1.50, 1.91)*** |
| undefined | 1.70 (1.30, 2.23)*** | 1.46 (1.22, 1.75)*** |
| wood plank | 1.87 (1.33, 2.63)*** | 1.29 (1.02, 1.63)* |
| wood with mud | 1.99 (1.82, 2.19)*** | 1.55 (1.46, 1.66)*** |
| Roof material, ref = ‘thatch’ |  |  |
| iron sheet | 3.21 (2.88, 3.57)*** | 1.44 (1.34, 1.55)*** |
| undefined | 1.44 (1.03, 2.02)* | 1.03 (0.80, 1.33) |
| Land cover, ref = ‘grasslands’ |  |  |
| croplands | 1.10 (0.83, 1.44) | 1.45 (1.18, 1.78)*** |
| savannas | 0.87 (0.80, 0.96)** | 0.91 (0.86, 0.97)** |
| urban and built-up lands | 1.08 (0.82, 1.42) | 1.25 (1.02, 1.53)* |
| woody savannas | 0.83 (0.74, 0.93)** | 0.83 (0.76, 0.90)*** |
| EVI | 1.07 (1.03, 1.11) *** | 1.09 (1.06, 1.12)*** |
| night-time LST | 0.82 (0.77, 0.86) *** | 0.82 (0.79, 0.85)*** |
| day-time LST | 1.00 (0.94, 1.06) | 0.98 (0.94, 1.03) |
| rainfall | 0.79 (0.72, 0.87)*** | 0.76 (0.71, 0.82)*** |

## **Appendix 1: ATSB station monitoring questionnaire.**

| **Question or prompt #** | **Question** | **Type of response** | **Options for response** |
| --- | --- | --- | --- |
| 1 | What type of visit are you making to this structure?  If you are conducting your normal visit schedule, select ‘Routine Monitoring’ regardless of the condition of the ATSB.     Only select ‘Responding to damage’ if a household has called you because their ATSB became damaged in between your normal visit schedule. | Choice | 1. Routine monitoring of bait station 2. Responding to damaged ATSB 3. Installing on new structure |
| 2 | Stand just outside the structure entrance, record location | GPS |  |
| 3 | How many bait stations are currently installed on this structure? | Integer |  |
| Prompt1  [if Q3 = 0] | If this is a **new structure** you need to exit and select ‘install on new structure' on the first question | Prompt |  |
| 4  [if Q3 =0] | Please explain reason for 0 ATSBs | Choice | 1. Weather 2. Someone took it down 3. Unknown 9. Other |
| 5  [if Q4 = 9] | Specify other reason for 0 ATSBs | Text |  |
|  | **ATSB 1 on the structure** | **Group** |  |
| 6  [if Q3>=1, otherwise skip to Prompt4]] | Scan barcode of **first** bait station **currently installed** on the structure | Barcode |  |
| 7 | Confirm that the barcode scanned matches the number appearing on the bait station's barcode | Choice | 1. Yes 0. No |
| 8  [if Q7 =0, or scan feature unresponsive] | Manually enter barcode of first bait **currently installed** station on the structure | Number |  |
| 9  [if Q8 has been answered] | Enter again the barcode of first bait **currently installed** station on the structure | Number |  |
| 10 | Please take a photo of bait station ____ | Image |  |
| 11 | Does bait station ____ have any holes, tears or punctures in any of the cells? | Choice | 0. No 1. Yes |
| 12  [if Q11 = 1] | On bait station ____ , are 1 or more cells completely torn open? | Choice | 0. No 1. Yes |
| 13 | Is bait station ____ leaking?  Leaking is defined as bait/liquid coming off the black membrane onto the white border or nearby surroundings (wall or ground).  Do not replace the ATSB if it is “sweating” or “sticky” | Choice | 0. No 1. Yes |
| 14  [if Q13=1] | Please take a photo of bait station ____ showing leakage  The photo should show the leakage dripping to the ground | Image |  |
| 15 | Does bait station ____ have any mold growth?  Mold is fuzzy growth on the surface of the membrane; mold often looks like white, black, or brown fuzz. | Choice | 0. No 1. Yes |
| 16  [if Q15=1] | What type of mold is on bait station ____ ?  (tick all that apply) | Choice | 1. Spots of mold on cells 2. Layer of black mold across bait station |
| 17  [if Q16=1] | On bait station ____ , **is any spot of mold larger than the rubber end of a pencil**?   This may include spots of mold that are ‘touching’ to form a group. | Choice | 0. No 1. Yes |
| 18  [if Q16=2] | On bait station ____ , **is the layer of black mold covering more than half of the bait station**? | Choice | 0. No 1. Yes |
| 19 | Is bait depleted from any of the cells on bait station ____ ? | Choice | 0. No 1. Yes, 1-7 cells are depleted of bait 2. Yes, 8 or more cells are depleted of bait |
| 20 | Is bait station ____ dirty? | Choice | 0. No 1. Yes, 1-7 cells are dirty 2. Yes, 8 or more cells are dirty |
| Prompt2  [if Q12=1, or Q13=1, or Q17=1, or Q18=1, or Q19=2, or Q20=2] | You need to remove bait station ____ and install a new bait station | Prompt |  |
| 21  [if Prompt2 is triggered] | CONFIRM: did you remove bait station ____ | Choice | 0. No 1. Yes |
| 22  [if Prompt2 is triggered] | CONFIRM: have you installed a new bait station? | Choice | 0. No 1. Yes |
| Prompt3  [if Q22=0] | Please install a new bait station to replace ____ | Prompt |  |
| Prompt4  [if Q3=0] | You need to install 2 bait stations on this structure | Prompt |  |
| Prompt5  [if Q3=1] | You indicated that there was **one bait station currently on this structure**. You need to install another so there are a total of 2 bait stations on this structure | Prompt |  |
| 23  [if Q22=1, or Prompt4 is triggered, or Prompt 5 is triggered] | Scan barcode of the **new** bait station in **position 1** on the structure | Barcode |  |
| 24  [if Q23 is triggered] | Confirm that the barcode scanned matches the number appearing on the bait station's barcode | Choice | 0. No 1. Yes |
| 25  [if Q24 =0, or scan feature unresponsive] | Manually enter barcode of the **new** bait station in **position 1** on the structure | Number |  |
| 26  [if Q25 has been answered] | Enter again the barcode of the **new** bait station in **position 1** on the structure | Number |  |
|  | **ATSB 2 on the structure** | **Group** |  |
| 27  [if Q3>1] | Scan barcode of **second** bait station **currently installed** on the structure | Barcode |  |
| 28 | Confirm that the barcode scanned matches the number appearing on the bait station's barcode | Choice | 0. No 1. Yes |
| 29  [if Q28 =0, or scan feature unresponsive] | Manually enter barcode of **second bait station currently installed** station on the structure | Number |  |
| 30  [if Q29 has been answered] | Enter again the barcode of **second bait station currently installed** station on the structure | Number |  |
| 31 | Please take a photo of bait station ____ | Image |  |
| 32 | Does bait station ____ have any holes, tears or punctures in any of the cells? | Choice | 0. No 1. Yes |
| 33  [if Q32 = 1] | On bait station____  , are 1 or more cells completely torn open? | Choice | 0. No 1. Yes |
| 34 | Is bait station ____ leaking?  Leaking is defined as bait/liquid coming off the black membrane onto the white border or nearby surroundings (wall or ground).  Do not replace the ATSB if it is “sweating” or “sticky” | Choice | 0. No 1. Yes |
| 35  [if Q34=1] | Please take a photo of bait station ____ showing leakage  The photo should show the leakage dripping to the ground | Image |  |
| 36 | Does bait station ____ have any mold growth?   Mold is fuzzy growth on the surface of the membrane; mold often looks like white, black, or brown fuzz. | Choice | 0. No mold 1. Mold on 1 or more cells |
| 37  [if Q36=1] | What type of mold is on bait station ____ ?  (tick all that apply) | Choice | 1. Spots of mold on cells 2. Layer of black mold across bait station |
| 38  [if Q37=1] | On bait station ____ , **is any spot of mold larger than the rubber end of a pencil?**  This may include spots of mold that are ‘touching’ to form a group. | Choice | 0. No 1. Yes |
| 39  [if Q37=2] | On bait station ____ , **is the layer of black mold covering more than half of the bait station**? | Choice | 0. No 1. Yes |
| 40 | Is bait depleted from any of the cells on bait station ____ ? | Choice | 0. No 1. Yes, 1-7 cells are depleted of bait 2. Yes, 8 or more cells are depleted of bait |
| 41 | Is bait station ____ dirty? | Choice | 0. No 1. Yes, 1-7 cells are dirty 2. Yes, 8 or more cells are dirty |
| Prompt6  [if Q33=1, or Q34=1, or Q38=1, or Q39=1, or Q40=2, or Q41=2] | You need to remove bait station ____ and install a new bait station | Prompt |  |
| 42  [if Prompt6 is triggered] | CONFIRM: did you remove bait station ____ | Choice | 0. No 1. Yes |
| 43  [if Prompt6 is triggered] | CONFIRM: have you installed a new bait station? | Choice | 0. No 1. Yes |
| Prompt7  [if Q43=0] | Please install a new bait station to replace ____ | Prompt |  |
| 44  [if Q43=1, or Prompt4 is triggered, or Prompt 7 is triggered] | Scan barcode of the **new** bait station in **position 2** on the structure | Barcode |  |
| 45  [if Q44 is triggered] | Confirm that the barcode scanned matches the number appearing on the bait station's barcode | Choice | 0. No 1. Yes |
| 46  [if Q45 =0, or scan feature unresponsive] | Manually enter barcode of the **new** bait station in **position 2** on the structure | Number |  |
| 47  [if Q46 has been answered] | Enter again the barcode of the **new** bait station in **position 2** on the structure | Number |  |
| ***Provisions were made to collect data on any extra ATSB stations found on a structure during monitoring, up to the 5^th^ ATSB station.***  ***[If Q3 >2, Q27-42 would repeat for each extra ATSB station in turn. Extra ATSB stations were not replaced if found to be damaged]*** | | | |
|  | **End of form** | **Group** |  |
| Prompt8  [if Q3>2] | A structure should have a maximum of 2 bait stations.  Were some bait stations moved from other structures? If so move them back to the original location. | Prompt |  |
| 48  [if Prompt8 is triggered] | What action did you take for the **extra bait stations** at this structure? |  | 1. Returned to original structure 2. Removed and discarded in waste 3. Removed and kept for future installation elsewhere 4. Left hanging on the structure 9. Other action |
| 49  [if Q48=9] | Specify other action you have taken for the **extra bait stations** on this structure | Text |  |
| 50 | Please enter any additional comments | Text |  |

## **Appendix 2: Additional structure-level information collected during the removal campaign.**

| **Question or prompt #** | **Question** | **Type of response** | **Options for response** |
| --- | --- | --- | --- |
| 1 | What is the main material of the wall of this structure? | Choice | 1. Grass or thatch  2. Wood with mud  3. Mud brick  4. Cement  5. Wood plank  96. Other |
| 2  [If Q1=96] | Describe the wall material | Text |  |
| 3 | What is the main material of the roof of this structure? | Choice | 1. Thatch  2. Iron sheet  3. Other |
| 4  [If Q3=96] | Describe the roof material | Text |  |
| 5 | Take a photo that captures the entire structure.   Include the exterior wall, roof, and ground in the photo. You should include a wall that has a bait station installed | Image |  |
|  | **ATSB 1** | **Group** |  |
| 6 | What level of protection does the **first** bait station on the structure have from sunlight or rain? | Choice | 1. Well protected - Bait station is tucked under the roof so that you cannot see the top bamboo stick OR it is under a large veranda area  2. Slightly protected - Roof overhang provides some shade, but not full protection of the bait station. Bait station is still near to the top of the structure.  3. Little or no protection- Roof does not extend away from the wall, there is no shade for the bait station OR bait station is installed halfway down the wall |
|  | **ATSB 2** | **Group** |  |
| 7 | What level of protection does the **second** bait station on the structure have from sunlight or rain? | Choice | 1. Well protected - Bait station is tucked under the roof so that you cannot see the top bamboo stick OR it is under a large veranda area  2. Slightly protected - Roof overhang provides some shade, but not full protection of the bait station. Bait station is still near to the top of the structure.  3. Little or no protection- Roof does not extend away from the wall, there is no shade for the bait station OR bait station is installed halfway down the wall |
